# Evaluating the antimicrobial effect, compressive strength and fluoride release of glass ionomer cement modified with Triphala

**DOI:** 10.1038/s41598-025-23936-6

**Published:** 2025-11-10

**Authors:** Yasmine Mohamed Afify, Gehan Gaber Allam, Ola Abd El-Geleel

**Affiliations:** 1 Pediatric Dentistry and Dental Public Health Department, Faculty of Dentistry, Ain Shams University, Organization of African Unity St, El-Qobba Bridge, El Weili, Cairo, Egypt; 2 Faculty of Dentistry, British University in Egypt, Suez Desert Road , Cairo 11837 - P.O. Box 43, El Sherouk City, Egypt

**Keywords:** Compressive strength, Fluoride release, Antimicrobial effect, Triphala, Health care, Materials science, Medical research, Microbiology

## Abstract

The enhancement of the antibacterial properties of glass ionomer cement has gained substantial importance in contemporary restorative dentistry, as microbial biofilms are the primary cause of secondary caries and restoration failure. Recent advances focus on modifying glass ionomer cement by incorporating antimicrobial agents to significantly improve its antibacterial activity. Therefore, this study aims to evaluate the antimicrobial effect, compressive strength, and fluoride release of glass ionomer cement modified with Triphala. This in vitro study included a total of 78 glass ionomer specimens, which were allocated into three primary testing categories: antibacterial activity, compressive strength, and fluoride release. Specifically, (*n* = 24) specimens were designated for the antibacterial test, (*n* = 33) specimens for evaluating compressive strength, and (*n* = 21) specimens for assessing fluoride release. Each testing category was further subdivided into three groups based on the incorporation of Triphala into the glass ionomer cement. Group A served as the control, consisting of glass ionomer without Triphala, Group B included glass ionomer specimens modified with 1.25%wt Triphala, and Group C comprised glass ionomer specimens modified with 3%wt Triphala. There was a significant difference among the three groups in terms of antibacterial activity and fluoride release tests, with Group C exhibiting the highest values in both tests. However, no significant difference was reported between the mean compressive strength of the control group and the experimental groups. Results revealed that Triphala enhances the antibacterial and fluoride-releasing abilities without compromising the compressive strength of GIC.

## Introduction

Glass ionomer cement (GIC) was first developed by Wilson and Kent in 1969 at the Government Chemist Laboratory in London, United Kingdom, and was widely utilized in the dental field as a luting agent, cavity liner, base, and restorative material due to its unique chemical adhesion to tooth structure and fluoride-releasing properties^1^. Despite these advantageous features, GIC exhibit several notable limitations as their mechanical properties are relatively poor compared to other dental materials, which are characterized by low compressive strength and limited abrasion resistance. In addition, they demonstrate limited bactericidal effects, reducing their antimicrobial efficacy^2^. To overcome these drawbacks, various modifications have been explored to enhance the mechanical and antibacterial properties of conventional GIC by the incorporation of new materials, such as E-glass fiber^3^, silver-doped nanotube fillers^4^, and herbal extracts, such as Triphala, with different approaches^5,6^.

Triphala is a renowned powdered herbal formulation that has been traditionally used since ancient times in the Indian System of Medicine. It is composed of three individual plants: Terminalia chebula, Terminalia belerica, and Emblica officinalis, and these components are combined in equal proportions to form the Triphala mixture. Triphala has demonstrated significant antibacterial and anti-caries properties relevant to oral health^7^. Specifically, Terminalia chebula plays a crucial role in preventing and treating various oral conditions such as stomatitis, gingivitis, dental caries, and bleeding gums. The extract of Terminalia chebula effectively inhibits plaque formation on the tooth surface by preventing sucrose-induced adherence and glucan-induced aggregation, which are two key processes that facilitate the colonization of cariogenic bacteria as *Streptococcus mutans*^8^.

Previous studies had demonstrated the efficacy of Triphala in dentistry, highlighting its antimicrobial properties without adverse effects on oral health. A notable clinical study was conducted by Paulra et al. (2020)^9^, to evaluate the antimicrobial activity of glass ionomer cement (GIC) modified with Triphala at a ratio of 1:0.5:0.5 (powder GIC: liquid GIC: Triphala extract) and 50% propolis against *Streptococcus mutans* and *Lactobacillus Acidophilus*. Modified GIC groups with Triphala exhibited significantly larger zones of inhibition compared to the control, confirming that GIC modified with Triphala possesses strong antimicrobial properties.

In addition, another clinical study conducted by Patel et al. **(**2024)^10^, to assess the antibacterial efficacy of mouthwashes containing 0.6% Triphala, 0.3% asafoetida, and 0.2% chlorhexidine on the salivary load of *Streptococcus mutans* in children with active dental caries. The results demonstrated that both Triphala and asafoetida mouth rinses significantly reduced *Streptococcus mutans* levels, with Triphala exhibiting superior antimicrobial activity compared to asafoetida. Patel et al. concluded that herbal mouthwashes containing Triphala and asafoetida are effective natural alternatives for controlling cariogenic bacteria in saliva.

To the best of our knowledge, no study has specifically evaluated both the compressive strength and fluoride release of GIC modified with Triphala. Therefore, the objective of this in-vitro study was to evaluate the antibacterial effect, compressive strength, and fluoride release of conventional GIC modified with Triphala.

## Subject and methods

This study is an in vitro experimental study conducted at the Faculty of Dentistry, Ain Shamis University, Dental Materials Department. The Faculty of Pharmacy, British University in Egypt. The Faculty of Science, Ain Shamis University. The materials that have been used to conduct this research, their composition and manufactures’ details are listed in (Table 1).

**Table 1:** Materials used in the study, composition, and manufacture.

Material	Composition	Manufacturer
Glass ionomer restorative material	A. Powder composition: Fluorosilicate glass B. Liquid composition: • Polyacrylic acid • Water • Tartaric acid • Gluconodelta-lactone • Parabens	Medifil, Germany
Triphala	Amalaki, Bibhitaki, Haritaki	Biotiva, Germany
Muller-Hinton medium	• Beef extract 2.0G/L • Acid hydrolysate 17.5G/L • Starch 1.5 G/L • Agar 17.0 G/L	John Hinton, Massachusetts, United States
• *Streptococcus mutans* • *Lactobacillus Acidophilus*		Microbiologics, Inc., Minnesota, United States. • SM strain ATCC 10,449 • Lactobacillus Acidophilus strain ATCC 4356

### Sample size Estimation

A power analysis was designed to have adequate power to apply a statistical test of the null hypothesis that there is no difference would be found between different groups. The effect sizes used in the sample size calculation were derived from previous studies that employed similar experimental conditions and specimen preparation protocols^9,11^.

**Regarding the antibacterial test**: By adopting an alpha level of (0.05), a beta of (0.2), i.e., power = 80% and an effect size (f) of (0.680), calculated based on the findings of Paulraj, et al.^9^ The predicted sample size (n) was a total of (24) samples (i.e., 8 samples per group) **Regarding the compressive strength**: By adopting an alpha level of (0.05), a beta of (0.2), i.e., power = 80% and an effect size (f) of (0.568) calculated using the results of Allam, et al.^11^ The predicted sample size (n) was a total of (33) samples (i.e., 11 samples per group). **Regarding the fluoride release**: By adopting an alpha level of (0.05), a beta of (0.2), i.e., power = 80% and an effect size (f) of (0.599) calculated based on the findings of Allam, et al.^11^ The predicted sample size (n) was a total of (21) samples (i.e., 7 samples per group Sample size calculation was performed using G*Power version 3.1.9.7.

### Sample grouping

A total of 78 GIC specimens were used in this study, divided into three main testing groups: (*n* = 24) specimens for the antibacterial test, (*n* = 33) specimens for the compressive strength test, and (*n*= 21) specimens for the fluoride release test. Each of these testing groups was further subdivided into three subgroups as follows: Group A included glass ionomer without Triphala, Group B contained glass ionomer modified with 1.25%wt Triphala, and Group C comprised glass ionomer modified with 3%wt Triphala. All samples are assigned unique numbers, and these numbers are placed individually into opaque, sealed envelopes to maintain allocation concealment. This ensures that the assignment is blinded, preventing selection bias. Then, the computer software is used to generate a random sequence or list of numbers. The samples are allocated according to this randomized sequence by matching the random numbers with the sample numbers within the envelopes^12^. A double-blind approach was used in this study, where the outcome assessor as well as the statistician were blinded to the group allocation by coding the samples.

A pilot study was conducted in which 1.25%, 3%, and 5% wt Triphala were tested. These concentrations were selected in accordance with prior similar studies^11,13^ that showed effective antibacterial activity and satisfactory mechanical performance at these levels, ensuring comparability and relevance to existing literature. According to the pilot results, 1.25% and 3% Triphala concentrations enhanced the antibacterial potency of glass ionomer cement without compromising its mechanical properties, whereas 5% Triphala adversely affected mechanical strength.

### Preparation of specimens

The GIC specimens were prepared as follows: for Group A, Glass ionomer powder was mixed with liquid according to the manufacturer’s instructions at a ratio of 1:1. In Group B, the experimental powder was formulated by incorporating 1.25wt% Triphala into the GIC powder using a digital balance to ensure precise measurement. To ensure accurate incorporation of Triphala powder into the glass ionomer cement powder, geometric dilution was employed during mixing, guaranteeing the homogenous distribution of Triphala, followed by mixing with the glass ionomer liquid. Similarly, in Group C, the experimental powder was prepared by blending 3wt% Triphala with the GIC powder using a digital balance, and then mixed with the glass ionomer liquid. To ensure accurate incorporation of Triphala into the glass ionomer cement powder, geometric dilution was employed during mixing, guaranteeing the homogenous distribution^14^.

### Study procedure

#### Antibacterial test

##### Specimens Preparation

Disc-shaped GIC specimens were fabricated using a split Teflon mold with dimensions of 3 mm in height and 6 mm in diameter^15^. Each experimental material was dispensed onto a mixing pad and mixed for 30 s with a sterile plastic spatula to obtain a homogenous mix. The mixed material was then carefully placed into the mold, and the top of each specimen was covered by a celluloid strip. A glass slide with a load of 1 kg was applied to achieve a smooth, flat surface and consistent specimen thickness^16^. The specimens were gently removed from the mold, and any excess or flash material around the edges was meticulously polished using surgical scalpels to obtain uniform and standardized specimen dimensions^17^.

***Streptococcus mutans*** **(SM) and** ***Lactobacillus Acidophilus*** **culture**: The antimicrobial efficacy of the tested material was assessed against standard strains of *SM* and *Lactobacillus Acidophilus*. The sources for *SM* and *Lactobacillus*bacteria are typically standardized microbial strains obtained from the American Type Culture Collection (ATCC). S.M strain ATCC 10,449 and Lactobacillus acidophilus strain ATCC 4356 were used^18^. *SM* was dispersed on Mitis salivarius bacitracin (MSB) agar, whereas *Lactobacillus acidophilus*was dispersed on de Man, Rogosa, and Sharpe (MRS) agar. The two plates were incubated at 37°c for twenty-four hours to obtain a culture of SM and Lactobacillus^19^. After 24 h, colonies of SM and Lactobacillus were obtained and then plated onto Mueller-Hinton agar separately using a transport swab stick^20^.

**Fig. 1:**
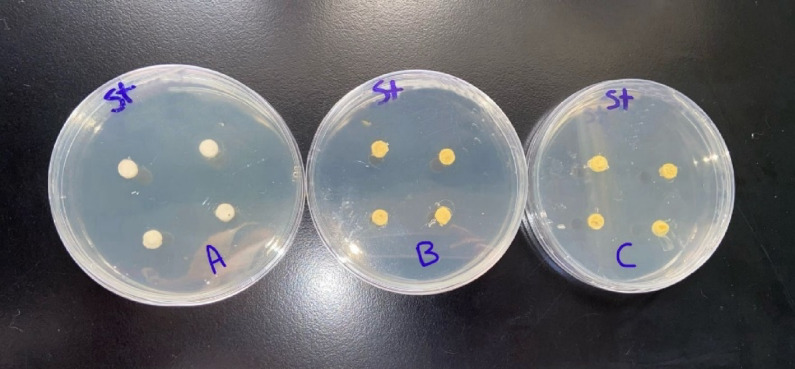
GIC specimens embedded in agar plates of Group A, Group B, and Group C were inoculated with *Streptococcus mutans* bacteria.

**Fig. 2:**
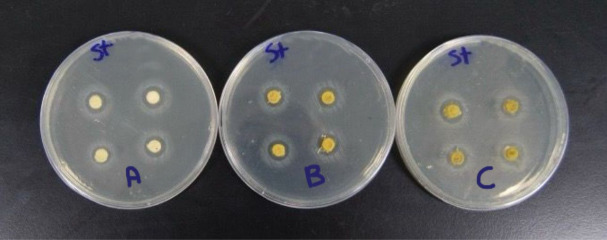
GIC specimens embedded in agar plates of Group a, Group b, and Group c were inoculated with *Lactobacillus Acidophilus* bacteria.

**Fig. 3:**
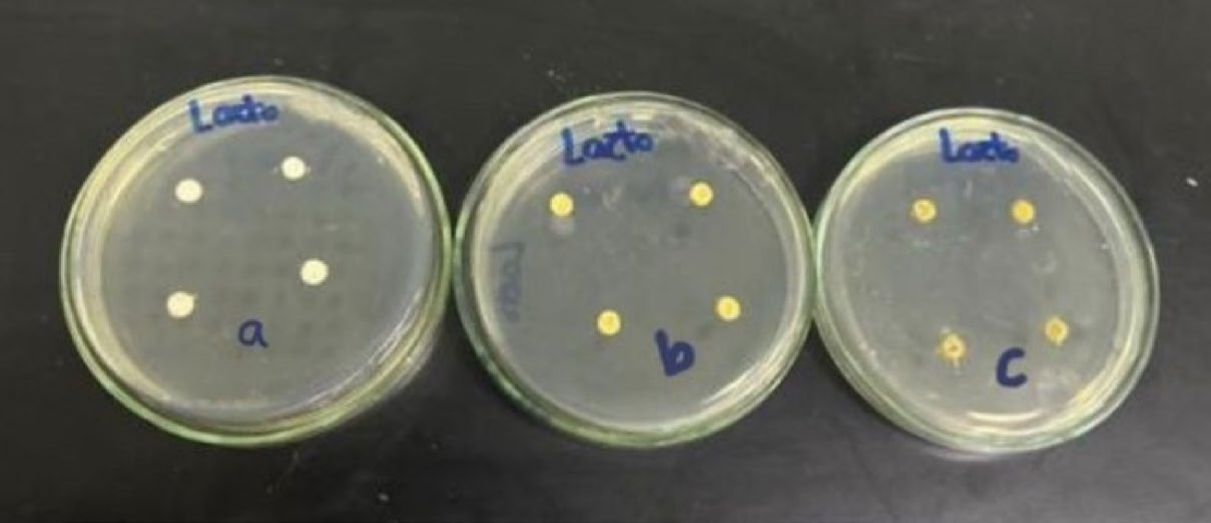
Incubated agar plates showing inhibition zones surrounding the GIC specimens of Group A, Group B, and Group C were inoculated with *Streptococcus mutans* bacteria.

**Fig. 4:**
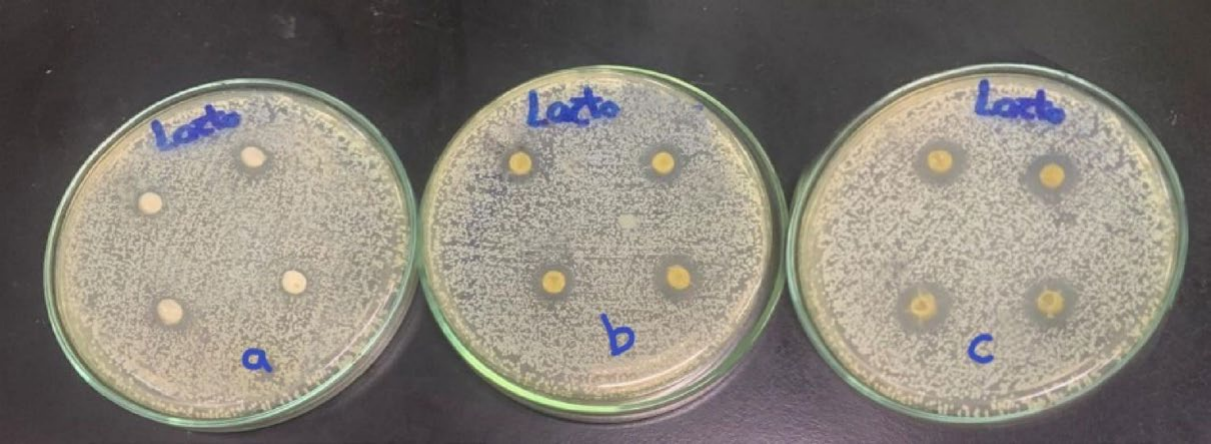
Incubated agar plates showing inhibition zones surrounding the GIC specimens of Group a, Group b, and Group c were inoculated with *Lactobacillus Acidophilus* bacteria. “Groups labeled with capital letters A, B, and C correspond to specimens that were inoculated with *Streptococcus mutans*, while groups labeled with lowercase letters a, b, and c correspond to specimens that were inoculated with *Lactobacillus Acidophilus*.*”*.

**Agar diffusion disc test**: The agar diffusion test was carried out on six agar plates of Mueller-Hinton medium that was prepared according to standardized protocols. Specifically, 38 g of Mueller-Hinton agar powder was suspended in 1000 mL of distilled water and boiled to ensure complete dissolution of the medium. The solution was then sterilized by autoclaving at 15 Ibs (121 °C) for 15 min^21^. Four disc-shaped specimens were placed on the surface of each agar plate for every experimental group. For each group, two plates were prepared: one plate was inoculated with Streptococcus mutans (Fig. 1), and the other with Lactobacillus Acidophilus (Fig. 2). The three experimental groups consisted of Group A (control: glass ionomer cement without Triphala), Group B (GIC modified with 1.25%wt Triphala), and Group C (GIC modified with 3%wt Triphala). This setup resulted in a total of eight specimens tested per group across both bacterial strains^22^. The plates were incubated at 37 °C for 48 h in an incubator. Following the incubation period, the plates were examined for the presence of inhibition zones surrounding each specimen (Figs. 3 and 4). The diameter of the inhibition zones was measured in centimeters using a plastic ruler^23^.

#### Compressive strength test

##### Specimens Preparation

Eleven cylindrical specimens were prepared for each experimental group using prefabricated Teflon molds with dimensions of 6 mm in height and 4 mm in diameter^24^. The specimens were removed from the Teflon molds immediately after the initial setting phase, which typically occurs within a few minutes (approximately 2–6 min) after mixing due to the acid-base reaction characteristic of GIC. Upon removal from the molds, all specimens were immersed in deionized water and stored at room temperature for one week to allow for further maturation processes, including continued ion crosslinking and hydration, which enhance the mechanical properties and stability of the cement^25^.

**Mechanical Testing Procedure**: Compressive strength testing was conducted in accordance with the ISO 9917−1:2007 standard^26^. All tests were performed at ambient laboratory conditions, maintained at 23 ± 1 °C. Each specimen was positioned in a universal mechanical testing machine (Instron, Lloyd, UK), and a uniaxial compressive load was applied at a constant crosshead speed of 1 mm/min until catastrophic failure of the specimen occurred^27^.

**Calculation of compressive strength**: The compressive strength was subsequently calculated using the following formula: *CS* = *πD*24*F*, where CS represents the compressive strength in megapascals (MPa), F is the maximum load at fracture in Newtons, and D is the diameter of the specimen in millimeters (approximately 4 mm). The results were reported as mean compressive strength values with corresponding standard deviations to assess the mechanical performance of the tested GIC materials^28^.

#### Fluoride release test

##### Specimen Preparation

Seven disc-shaped specimens were fabricated using prefabricated Teflon molds with dimensions of 3 mm in height and 6 mm in diameter^15^. Following fabrication, the specimens were incubated at 37 °C for 24 h to ensure complete setting before testing^29^. Each specimen was then individually immersed in 20 mL of deionized water within a sealed plastic container. After one week from the start of the experiment, the specimens were removed, and the storage solutions were collected for the measurement of fluoride ion release^30^. Subsequently, the specimens were gently dried and transferred to fresh containers containing 20 mL of deionized water to measure fluoride ion release after one month^31^. Fluoride ion concentration was measured using ion chromatography (Thermo ICS1100, USA) at the Faculty of Science, Ain Shams University. The results were expressed in parts per million (ppm)^32^.

**Statistical analysis** was done using IBM SPSS^®^ Statistics version 26 (IBM^®^ Corp., Armonk, NY, USA). Numerical data were expressed as mean and standard deviation or median and range as appropriate. Data were tested for normality using the Kolmogorov-Smirnov test and the Shapiro-Wilk test. Comparison between 3 groups was done using either Analysis of Variance (ANOVA) or Kruskal-Wallis test (non-parametric ANOVA), then a post-Hoc test was used for pair-wise comparison. Comparison between two consecutive measures of numerical variables was done using a paired t-test. All tests were two-tailed. A p-value < 0.05 was considered significant.

## Results

1. **Antibacterial testing**: Intergroup comparison and summary statistics for the antibacterial test are represented in Tables 2 and 3.

### A. Streptococcus mutans (SM) Inhibition

There was a significant difference between the three groups in the SM inhibition zone (*p* = 0.026). The largest inhibition zone was found in Group C (1.2 cm [1.2–1.3]), followed by Group B (1.1 cm [1.0–1.1.0.1]), while the smallest inhibition zone was found in Group A (1.0 cm [0.9–1.0.9.0]). Post-hoc pairwise comparisons showed a significantly larger inhibition zone in Group C compared to Group A (*p* = 0.006).

**Table 2:** Intergroup comparison and summary statistics for S.M Inhibition zone (in centimeters).

Groups	Median (Range)	*p*-value*
Group A	1.0 (0.9–1.0.9.0)^a^	0.008*
Group B	1.1 (1.0–1.1.0.1)^ab^	
Group C	1.2 (1.2–1.3)^b^	

Groups with **different superscript letters** are significantly different. *; significant (p<0.05).

### B. Lactobacillus acidophilus Inhibition

There was a significant difference between the three groups’ inhibition zone (*p* = 0.014). The largest inhibition zone was found in Group C (1.2 cm [1.1–1.2]), followed by Group B (1.1 cm [1.0–1.1.0.1]), while the smallest inhibition zone was found in Group A (1.0 cm [0.9–1.0.9.0]). Post-hoc pairwise comparisons showed a significantly larger inhibition zone in Group C compared to Group A (*p* = 0.012).

**Table 3:** Intergroup comparison and summary statistics for *Lactobacillus acidophilus* Inhibition zone (in centimeters).

Groups	Median (Range)	*p*-value*
Group A	1.0 (0.9–1.0.9.0)^a^	0.014*
Group B	1.1 (1.0–1.1.0.1)^ab^	
Group C	1.2 (1.1–1.2)^b^	

Groups with **different superscript letters** are significantly different. *; significant (p<0.05).

**2. Compressive strength**: Intergroup comparison and summary statistics for compressive strength are shown in Table 4. There was no significant difference between the three groups (*p* = 0.229). However, the compressive strength was relatively higher in Group B compared to Group C and Group A.

**Table 4:** Intergroup comparison and summary statistics for compressive strength (in MPa).

Groups	Mean ± SD	(Range)	*p*-value
Group A	57.5 ± 7.6	(45.5–70.3)	0.229
Group B	63.3 ± 9.9	(46.4–80.2)	
Group C	58.8 ± 6.4	(48.2–65.6)	

**3. Fluoride release**: Intergroup comparisons and summary statistics for fluoride release are shown in Table 5.

- Intergroup Comparison After 1 week.

There was a significant difference between the three groups in fluoride release (*p* = 0.008). The highest release was found in Group C (6.66 ± 1.36 mg/cm^2^), followed by Group A (6.25 ± 0.68 mg/cm^2^), while the lowest release was found in Group B (5.00 ± 0.38 mg/cm^2^). Post-hoc pairwise comparisons showed a significantly lower release in the compared to the Group C (*p* = 0.008), and the Group A (*p* = 0.047).

**Table 5:** Intergroup comparison and summary statistics for fluoride release after 1 week.

Groups	Mean ± SD	*p*-value*
Group A	6.25 ± 0.68^a^	0.014*
Group B	5.00 ± 0.38^b^	
Group C	6.66 ± 1.36^a^	

Groups with **different superscript letters** are significantly different. *; significant (p<0.05).

## Discussion

There is a paradigm shift toward the use of herbal extracts in dental research and clinical practice for the prevention and management of dental caries and other oral diseases. This shift is largely driven by the recognized medicinal qualities and potential advantages these natural agents offer for individuals^33^. Herbal plants have been shown to provide multiple oral health benefits, including the reduction of gingival inflammation, management of aphthous ulcers, antibacterial, antiviral, and anti-inflammatory effects^34^. An example of these herbal extracts used in oral health care is Triphala.

Triphala is a traditional powdered mixture of three herbal plants in the Indian system of medicine, which is well-recognized for its antimicrobial efficacy against dental caries. One of its key ingredients is Terminalia chebula, which acts as an anti-caries agent by inhibiting the adherence of *Streptococcus mutans*, thereby reducing biofilm formation and acid production on tooth surfaces^35^. Additionally, Terminalia bellirica, another component of Triphala, possesses tannic acid, which can bind to bacterial cell surfaces, causing protein denaturation and ultimately leading to bacterial cellular death^36^. The concentrations 1.25% wt and 3% wt Triphala were selected according to the pilot study results. The results confirmed that while 1.25% and 3% wt Triphala enhanced the antibacterial properties of GIC without detriment to mechanical strength.

Glass ionomer cement (GIC) was chosen as the test material for the current study due to its numerous advantages, including a true chemical bond to tooth structure, fluoride release that promotes remineralization, and excellent biocompatibility^37^. Nonetheless, conventional GICs have insignificant bactericidal potential and antibacterial activity against a limited range of pathogens^18^.

Compressive strength is a crucial property of GIC because it ensures the material can withstand the forces exerted during mastication without breakage or deformation. Since most masticatory forces are compressive in nature, evaluating the compressive strength of restorative materials like GIC is essential to predict their clinical performance and durability^38^.

Currently, there is a lack of studies investigating the effect of Triphala extract on the intrinsic mechanical properties and fluoride release of conventional GIC. Therefore, the primary objective of the present study was to evaluate the antimicrobial efficacy, mechanical properties, and fluoride release profile of GIC modified with Triphala extract. The current study aims to determine whether incorporating Triphala extract into conventional GIC could enhance its antibacterial potential without compromising its structural integrity and fluoride ion release, which are critical factors for its clinical performance.

The results of the current study showed that adding Triphala to glass ionomer significantly increased its antibacterial properties. As GIC modified with Triphala (3%wt) powder showed the superior inhibition, followed by GIC modified with Triphala (1.25%wt), while the smallest inhibition zone was found in the control group against both SM and Lactobacillus. These results are consistent with those of **Deshpande** et al. (2021)^39^, as the researchers carried out a clinical study to evaluate the effect of Triphala-based tooth-wipes on reducing salivary S.M and dental plaque in children with mild intellectual disability. The results demonstrated that the Triphala group presented a statistically significant reduction in SM counts after both 48 h and 7 days compared to baseline, and the presence of Triphala was crucial for a targeted antimicrobial effect against SM.

As previously mentioned, no study has specifically evaluated both the compressive strength and fluoride release of GIC modified with Triphala. Therefore, the results of the current study were compared with those of other studies involving GIC modified with other natural extracts (Salvia officinalis, miswak), providing a comprehensive evaluation of the effects of various natural compounds on the compressive strength and fluoride release of GIC.

Concerning the results of the compressive strength test, no significant difference was found between the control group and experimental groups (GIC modified with 1.25%wt and 3%wt T powder into the GIC matrix likely did not alter the fundamental chemical composition or the setting reaction of the cement. Therefore, modifying the GIC with Triphala powder didn’t affect the compressive strength of GIC.

The results of the present study coincide with those of **Forouzanmehr** et al. (2020)^40^, as they reported that the addition of Salvia officinalis plant extract to resin-modified glass ionomer cement didn’t compromise the compressive strength and shear bond strength of resin-modified glass ionomer. Similarly, **Devi** et al. (2024)^41^ concluded that adding plant extracts (Chirayita and Terminalia arjuna) with three different concentrations to conventional GIC showed no significant changes in the compressive strength of conventional GIC. These findings can be justified by the fact that the chemical compatibility and proper dispersion of these extracts within the GIC matrix prevent the formation of structural defects or weak points, thereby preserving the material’s integrity. On the contrary, incorporation of new materials such as E-glass fiber and silver-doped nanotube fillers were reported to enhance the compressive strength of GIC. Studies show that E-glass fiber, particularly at 20 wt%, significantly improves compressive strength, flexural strength, and hardness, while also reducing the solubility of GIC. ^*3*^Silver-doped nanotube fillers similarly enhance mechanical properties and provide antimicrobial benefits^4^.

The results of the current study revealed that there was a significant difference among the three groups in the fluoride release. The highest fluoride release was found in GIC modified with 3%wt Triphala, followed by the control group, while the lowest fluoride release was found in GIC modified with 1.25%wt Triphala. This reflects the ability of Triphala to potentiate the fluoride release ability. This finding was found to be in accordance with the study of **El Gamily** et al. (2018)^42^, which reported that incorporating miswak into glass ionomer cement enhanced its fluoride release ability. It was stated that the improvement can be attributed to various organic compounds that are present in miswak, which influence the setting reaction and porosity of GIC, potentially creating more pathways for fluoride ions to diffuse out of the material.

The major strength of the current study is the use of Triphala, which is a natural plant-based formulation renowned for its safety and biocompatibility. Unlike many synthetic agents, Triphala is nontoxic and well-tolerated, minimizing the risk of adverse effects, which is a critical consideration in clinical applications. Furthermore, Triphala is affordable and widely accessible, making it a cost-effective option for broader use, especially in resource-limited settings.

However, it is necessary to highlight some limitations; for instance, the in vitro nature of the study typically doesn’t replicate the oral environment. The study does not investigate the effects of Triphala addition on the setting time and solubility of GIC. Also, the incorporation of Triphala caused a darkening effect on the glass ionomer’s color.

Based on the findings of this study, it is recommended that future research explore a wider concentration range of Triphala incorporation into glass ionomer cement to establish a comprehensive dose-response relationship. Additionally, further investigations should assess other important physico-mechanical properties, including setting time, solubility, flexural strength, and wear resistance. In vivo studies are also essential to validate the antibacterial efficacy and fluoride release observed in vitro and to determine the long-term performance of GIC modified with Triphala in the oral environment.

## Conclusions

GIC modified with 3%wt Triphala showed the highest antibacterial efficacy and fluoride release, while the control had the least effect. No significant difference in compressive strength was observed among groups, though 1.25%wt Triphala-modified GIC demonstrated slightly higher strength. These findings suggest Triphala enhances antibacterial and fluoride-releasing properties without compromising mechanical integrity.

## Data Availability

Authors confirm that the datasets generated and/or analyzed during the current study are available from the first author, “Yasmine Afify,” upon request.

## Declarations

### Clinical trial

Not applicable as it is an in vitro experimental study.
